# Concurrent inhibition of enzymatic activity and NF-Y-mediated transcription of Topoisomerase-II*α* by *bis*-DemethoxyCurcumin in cancer cells

**DOI:** 10.1038/cddis.2013.287

**Published:** 2013-08-08

**Authors:** S Belluti, V Basile, P Benatti, E Ferrari, G Marverti, C Imbriano

**Affiliations:** 1 Dipartimento di Scienze della Vita, Università di Modena e Reggio Emilia, via Campi 213/D, Modena 41125, Italy; 2 Dipartimento di Scienze Chimiche e Geologiche, via Campi 283, Modena 41125, Italy; 3 Dipartimento di Scienze Biomediche, Metaboliche e Neuroscienze, via Campi 287, Modena 41125, Italy

**Keywords:** NF-Y, CCAAT box, gene transcription, TOP2A, curcuminoids

## Abstract

Topoisomerases-II*α* (TOP2A) enzyme is essential for cell viability due to its fundamental role in DNA metabolism and in chromatin organization during interphase and mitosis. TOP2A expression is finely regulated at the transcriptional level through the binding of the CCAAT-transcription factor NF-Y to its promoter. Overexpression and/or amplification of TOP2A have been observed in many types of cancers. For this reason, TOP2A is the target of the most widely successful drugs in cancer chemotherapy, such as TOP2A poisons, which stabilize TOP2A-DNA cleavage complexes and create DSBs, leading to chromosome damage and cell death. We previously reported that the Curcumin-derivative *bis*-DemethoxyCurcumin (bDMC) is an anti-proliferative agent that inhibits cell growth by concomitant G1/S and G2/M arrest. Here we showed that bDMC irreversibly induces DSBs in cancer cells, but not in normal cells, by targeting TOP2A activity and expression. TOP2A ablation by siRNA corroborates its contribution to apoptosis induced by bDMC. Short-term exposure to bDMC induces retention of TOP2A-DNA intermediates, while longer exposure inhibits TOP2A transcription by affecting expression and sub-cellular localization of NF-Y subunits. ChIP analysis highlighted reduced recruitment of NF-Y to TOP2A regulatory regions, concomitantly to histone deacetylation and decreased gene transcription. Our findings suggest that the dual activity of bDMC on TOP2A represents a novel therapeutic strategy to induce persistent apoptosis in cancer cells and identify NF-Y regulation as a promising approach in anti-cancer therapy.

Mammalian cells express two Topoisomerase II (TOP2) enzymes, TOP2A and TOP2B, but only the first is essential for cellular viability.^1, 2^ TOP2A has a key physiological function in DNA metabolism of eukaryotic cells and has a structural role in chromatin organization during interphase and mitosis.^3, 4^ The development of TOP2A-depleted mice terminates at the four- or eight-cell stage because of chromosome segregation defects.^5^ TOP2A expression is low in quiescent cells and increases when cells are stimulated to re-enter the growth phase of the cell cycle: its levels rise throughout the S phase and peak during G2/M phase.^6, 7, 8^ The expression of human TOP2A mRNA is controlled by its promoter region, whose activity is regulated through cis-acting elements within the first 617 base pairs.^9^ Among multiple transcription factors controlling TOP2A transcription throughout the cell cycle, Sp and NF-Y have been shown to bind GC consensus sites and four inverted CCAAT boxes (ICB1–ICB4), respectively.^10, 11, 12^

NF-Y is a heterotrimeric complex composed of three conserved subunits, NF-YA, NF-YB and NF-YC.^13^ NF-YA interacts with the NF-YB/NF-YC heterodimer and determines specific NF-Y binding to the CCAAT box. Although NF-Y acts as a transcriptional activator of TOP2A, its binding to ICB2 has been described to downregulate the promoter activity by confluence arrest.^14^

TOP2A transcription can be inhibited by wt p53 and the disruption of ICBs abolishes the p53-dependent downregulation of TOP2A.^15, 16^ In addition, p53-independent mechanisms can lead to decreased TOP2A mRNA levels through reduced NF-Y binding to ICB1.^15^

Increased TOP2A protein levels have been detected in cancer cells compared with non-malignant cells, although a strong heterogeneity of TOP2A expression within different cancer cells has been observed.^17^ For this reason, TOP2A is the target for some of the most widely successful drugs used to treat human cancers.^18, 19^

Two different classes of drugs targeting TOP2A have been described: the first one, referred to as TOP2A poisons, increases the concentration of covalent enzyme-cleaved DNA complexes and generates DNA strand breaks. The second class is composed by catalytic TOP2 inhibitors, compounds that prevent TOP2 from carrying out its physiological enzymatic activity.^18^

TOP2-DNA covalent complexes have severe effects on cell proliferation and viability: TOP2 poisons block both replication and transcription processes and lead to the activation of apoptosis by inducing DNA strand breaks.^20, 21, 22, 23^

Although the great potential of TOP2 targeting drugs towards multiple type of human cancers, TOP2 poisons can lead to secondary malignancies. In particular, etoposide and teniposide administration in chemotherapy regimens have a high potential of generating translocations, responsible for secondary malignancies,^24, 25^ while anthracyclines generate free radicals in both normal and tumor tissues, leading to bone marrow and cardiac toxicity.^26, 27^

Curcumin, the pigment derived from the rhizome *Curcuma longa*, has demonstrated anticancer properties, attributed to its selective cell death-inducing ability in tumor cells.^28, 29, 30^ The apoptotic process seems to be initiated by DNA damage triggered by TOP2 poisoning.^31, 32^ Despite the possible side effects of Curcumin on fertility and immune functions,^33, 34, 35^ in the majority of normal and primary cells, it does not elicit a cytotoxic response.

We demonstrated that the Curcumin derivative bis-DemethoxyCurcumin (bDMC) is more stable in physiological condition than its lead compound, circumventing one of the most limiting facets of Curcumin in clinical applications.^36^ bDMC exhibits improved cytotoxicity in ovarian cancer cells and shows higher anti-oxidant, anti-mutagenic, anti-carcinogenic and anti-metastatic activities than Curcumin.^37, 38^ While Curcumin delays the mitotic exit as a consequence of microtubule poisoning, bDMC is able to induce a concomitant and long-lasting G1/S and mitotic cell cycle block in human colon cancer cells. Concurrently to the cell cycle impairment, bDMC activates a DNA-damage response, which can be responsible for the slower kinetic in re-entering the cell cycle after the release.^36^

Our present investigations focus on the activity of bDMC as DNA-damaging agent and inhibitor of TOP2A. We highlight that bDMC triggers DNA damage in cancer cells by targeting TOP2A, both through inhibition of its enzymatic activity and through repression of its NF-Y-dependent transcription.

## Results

### Effect of bDMC on proliferation and cell cycle distribution in cancer and normal human cells

We have already shown that bDMC treatment induces a concomitant G1/S and long-lasting mitotic arrest of the cell cycle of human colon cancer cells HCT116. In particular, the passage from early to late S is delayed when bDMC is added to synchronized G0/G1 cells.^36^

**Figure 1:**
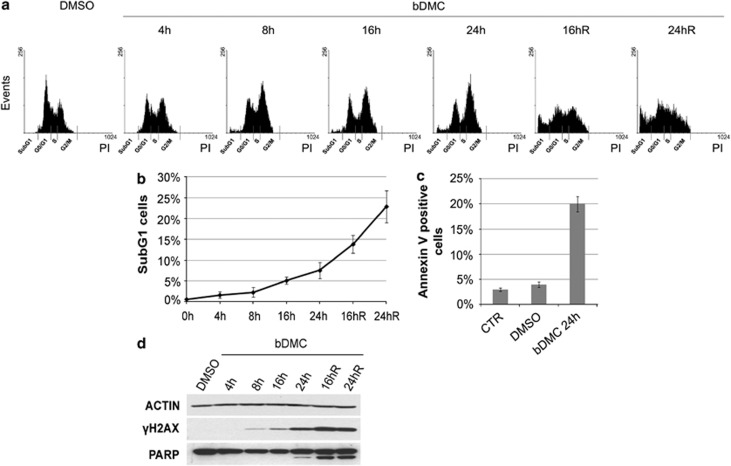
bDMC triggers irreversible cell death in HCT116 cells. (**a**) Cytometric cell-cycle analysis of HCT116 cells treated with 30 *μ*M bDMC for 4, 8, 16, 24 h and released in drug-free medium for additional 16 and 24 h (16hR and 24hR) following 24 h of bDMC treatment. (**b**) Percentages of SubG1 events detected by PI cytometric analysis following the indicated time exposure to bDMC. Reported values are an average of 10 independent experiments±S.D. (**c**) The percentage of Annexin V-positive cells after 24 h treatment with 30 *μ*M bDMC is compared with Control (CTR) and DMSO-treated cells. Data are means of three independent experiments±S.D. (**d**) Western blot expression analysis of *γ*H2AX and cleaved PARP1 following time-dependent administration of 30-*μ*M bDMC to HCT116 cells. Actin was used as loading control

In addition to cytostatic effects, cytotoxicity was observed following bDMC administration at IC50 concentration (30 *μ*M) to HCT116. A time-dependent increase of SubG1 events was detected by PI cytofluorimetric analysis in bDMC-treated cells (from 0.6% in control cells to 5.1 and 7.5% after 16 and 24 h, respectively) (Figures 1a and b). The presence of AnnexinV-positive cells (from 4% in dimethyl sulfoxide (DMSO) to 20% in bDMC cells) clearly suggested that bDMC triggers apoptotic cell death (Figure 1c). Interestingly, when HCT116 were released from bDMC into the fresh medium, SubG1 events raised up to about 14 and 23% after 16 and 24 h, hinting that bDMC irreversibly commits cells to apoptotic death (Figures 1a and b). Cleavage of PARP-1 and H2AX Ser139 phosphorylation (*γ*H2AX) followed the time-dependent increase of SubG1 events and further grew when cells were released from bDMC (Figure 1d). Exposure to bDMC induced the same effects on colorectal carcinoma Lovo cells (Supplementary Figures S1a–c).

**Figure 2:**
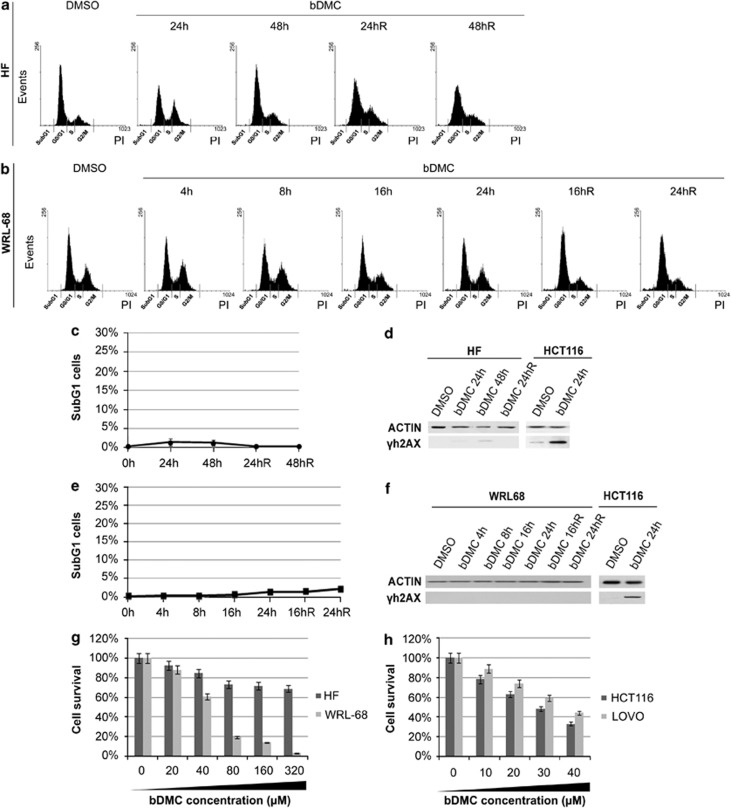
bDMC does not induce cell death in human healthy cells. (**a** and **b**) Cell-cycle analysis of HF (**a**) and WRL-68 (**b**) cells treated with 30 *μ*M bDMC for the indicated time points and released into fresh medium for additional 16, 24 or 48 h. (**c**) Quantification in percentage of SubG1 population in HF cells treated with bDMC and released into fresh medium for 24 and 48 h (24hR and 48hR). Values are means of three independent experiments±S.D. (**d**) Expression levels of *γ*H2AX in DMSO- and bDMC-treated and released (R) HF cells. (**e**) Percentage of SubG1 population in WRL-68 cells following bDMC administration and released into drug-free medium (16hR and 24hR). Values are means of three independent experiments±S.D. (**f**) Western blot analysis with anti-*γ*H2AX and anti-actin antibodies in WRL-68 cells treated and released from bDMC for the indicated time points. (**g** and **h**) Percentage of cell survival of HF and WRL-68 normal cells (**g**) and HCT116 and LOVO tumor cells (**h**) following administration with increasing concentrations of bDMC for 24 h

To investigate the cytotoxicity of bDMC against tumor *versus* healthy cells, human normal fibroblasts (HF) and hepatic fetal epithelial cells (WRL-68) were treated with 30 *μ*M bDMC (Figures 2a and b). No increase of SubG1 events was detected, also when cells were released from drug incubation (Figures 2c and e). The lack or very low expression of *γ*H2AX in treated and released cells further corroborates the tumor selectivity of bDMC (Figures 2d and f). Moreover, dose–response experiments highlighted that IC50 is significantly higher in HF (>320 *μ*M) and WRL68 (55 *μ*M) cells, compared with HCT116 and LOVO tumor cells (Figures 2g and h).

### bDMC induces double-strand breaks (DSBs) and chromosome aberrations in HCT116 cells

**Figure 3:**
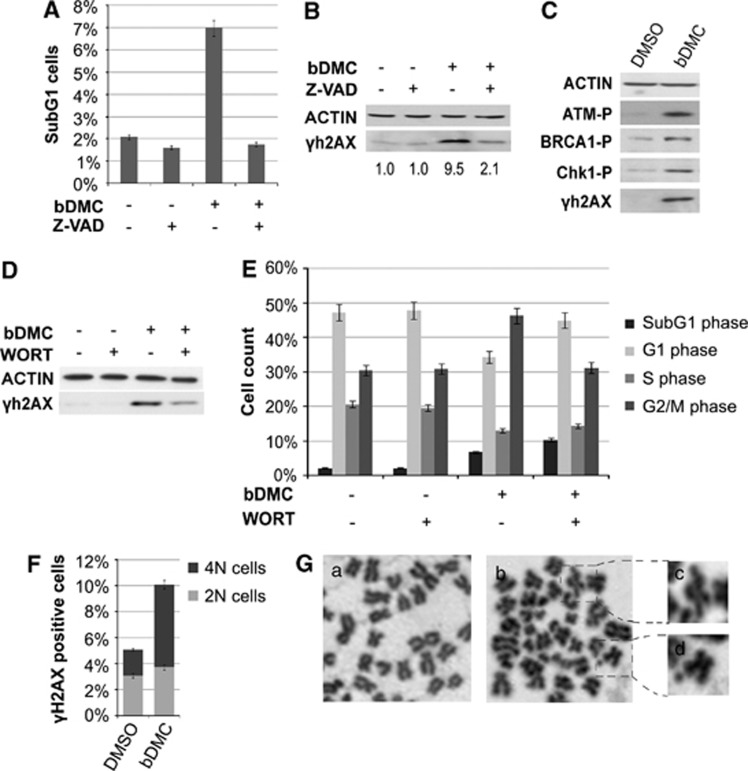
bDMC induces DNA damage and activates the DNA-damage response in HCT116 cells. (**A**) Quantification of SubG1 phase cell number following Z-VAD/bDMC co-treatment or bDMC (30 *μ*M) alone for 24 h. Data represent the average of three independent experiments±S.D. (**B**) Western blot and quantification of *γ*H2AX expression levels *versus* actin following bDMC and bDMC-Z-VAD 24-h treatments. (**C**) Western blot analysis of total extracts of 24-h 30-*μ*M bDMC-treated cells with the indicated antibodies. (**D**) Changes of *γ*H2AX expression levels in cells pre-treated with WORT compared with cells treated for 24 h with bDMC. (**E**) Percentage of cells throughout the different phases of the cell cycle of bDMC-treated cells before and after WORT pre-incubation. The indicated events are means of three independent experiments±S.D. (**F**) Percentages of *γ*H2AX-positive cells in 2N and 4N populations after 30-*μ*M bDMC treatments for 24 h. (**G**) Representative Giemsa*-*stained mitotic chromosome spread of cells treated with 30 *μ*M DMSO (a) or bDMC (b) for 24 h. Enlargement of dashed box (c and d) shows chromosome aberrations of bDMC-cells

H2AX phosphorylation has an important role not only in apoptosis but also in response to DSBs.^39^ To examine the relationship between *γ*H2AX and DNA damage rather than apoptosis, we co-treated cells with bDMC and the pan-caspase inhibitor Z-VAD. Although Z-VAD was able to completely abolish the accumulation of SubG1 events, *γ*H2AX was only partially reduced compared with bDMC-treated cells, coupling its expression to DNA damage (Figures 3A and B).

We previously demonstrated that bDMC administration for 24 h resulted in ATM (ataxia telangiectasia mutated) activation.^36^ ATM has a major role in signal transduction in response to DSBs,^40^ and once activated, it is able to phosphorylate different proteins associated with the DNA damage response, such as sensors, mediators, as H2AX and BRCA1, transducers and effectors of DSB, among which are ChK2, ChK1 and p53.^41^ Western blot analysis with Phospho-BRCA1 (Ser1524) and Phospho-ChK1 (Ser296) antibodies further demonstrated the activation of a DNA damage response induced by bDMC (Figure 3C). Pre-treatment with Wortmannin (WORT), which is able to disrupt multiple DNA damage and DNA repair mechanisms via inhibition of PI3K-kinases (ATM and DNA-PK),^42, 43, 44^ resulted in a clear reduction of H2AX phosphorylation (Figure 3D). In addition, WORT determined a robust decrease of G2/M population (from 46.3% in bDMC-treated cells to 31.2% in WORT-bDMC-treated cells) and an increase of G0/G1 events (from 34.4 to 44.9%), hinting that the activation of the DNA damage checkpoint occurs in G2/M and prevents cells from entering the next G1 phase (Figure 3E).

Fluorescent-activated cell sorting (FACS) was used to quantify the levels of H2AX phosphorylation in 2N and 4N populations. Following 24 h treatment with bDMC, we observed the accumulation of *γ*H2AX-positive cells with a 4N DNA content (Figure 3F), presumably G2/M arrested cells. Also HCT116/E6 cells, in which the lack of p53 causes a robust arrest in G2/M and prevents the G1/S block following bDMC administration,^36^ showed an evident time-dependent increase of *γ*H2AX, corroborating that DNA damaged cells are mainly distributed in the G2/M phase of the cell cycle (Supplementary Figures S1d and e).

We then evaluated the ability of bDMC to cause chromosome aberrations. Cells were treated with DMSO or bDMC for 24 h, and Giemsa-stained metaphases were analyzed: while control cells did not show chromosome abnormalities, we detected an average of two chromatid-type aberrations per cell, in particular gaps and breaks, following bDMC administration (Figure 3G). The same defects were detected in HCT116/E6 cells (data not shown).

All together these data suggest that bDMC induces DNA damage in G2/M cells, which are prevented from entering the next G1 phase and presumably are then committed to apoptosis.

### bDMC inhibits TOP2*α* activity

TOP1 and TOP2-DNA complexes have been observed following Curcumin administration to K562 cells.^32^ Considering that bDMC has a predicted stronger binding affinity to TOP2A compared with Curcumin,^45^ we hypothesized that DNA damage and chromosome aberrations induced by bDMC could be due to the inhibition of TOP2A in HCT116 cells.

**Figure 4:**
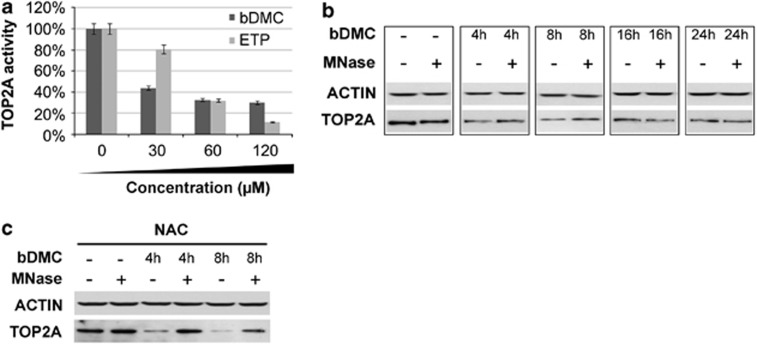
bDMC inhibits TOP2*α* enzymatic activity. (**a**) Inhibition of TOP2A-dependent decatenation of kDNA following increasing concentrations of bDMC and Etoposide (ETP) *versus* DMSO (arbitrarily set at 100%). (**b**) Western blot analysis of TOP2A expression after 4, 8, 16 and 24 h after 30 *μ*M bDMC addition, with or without MNase digestion. (**c**) TOP2A expression analysis of untreated and MNase-treated samples upon bDMC-NAC treatment

To test TOP2A inhibitory activity of bDMC, we performed a cell-free TOP2A relaxation assay (Figure 4a). The kinetoplast DNA (kDNA) was used as a specific substrate for TOP2 enzymes. In accordance with its known activity as TOP2A poison, etoposide inhibited TOP2A-dependent decatenation. bDMC was found to halved DNA decatenation at lower concentrations than etoposide.

To assess whether bDMC could act as TOP2A poison, we verified the formation of TOP2A-DNA complexes through a band-depletion assay.^46^ We compared the levels of TOP2A in the samples of each bDMC time point with or without Micrococcal Nuclease (MNase) addition, which allows the release of TOP2A from DNA complexes possibly induced by bDMC. MNase digestion partially restored TOP2A levels in cellular extracts from HCT116 treated with bDMC for 4 and 8 h, consistently with the presence of TOP2A-DNA complexes. Differently, no increase was observed following MNase addition in 16 and 24 h bDMC samples, hinting at a different mechanism triggering TOP2A depletion (Figure 4b).

Increase of cellular levels of reactive oxygen species (ROS) could have a role in the formation of TOP2-DNA complexes induced by Curcumin.^32^ Therefore, a band-depletion assay was performed with extracts from cells pre-treated with the antioxidant *N*-acetylcisteine (NAC). Figure 4c shows that NAC did not abolish TOP2A band depletion, ruling out that ROS could mediate TOP2A-DNA complexes.

These data clearly suggest that bDMC induced retention of TOP2A-DNA intermediates up to 8 h, after which reduced protein levels cannot be ascribed to bDMC activity as TOP2 poison.

### bDMC impairs NF-Y-dependent gene transcription of TOP2*α*

**Figure 5:**
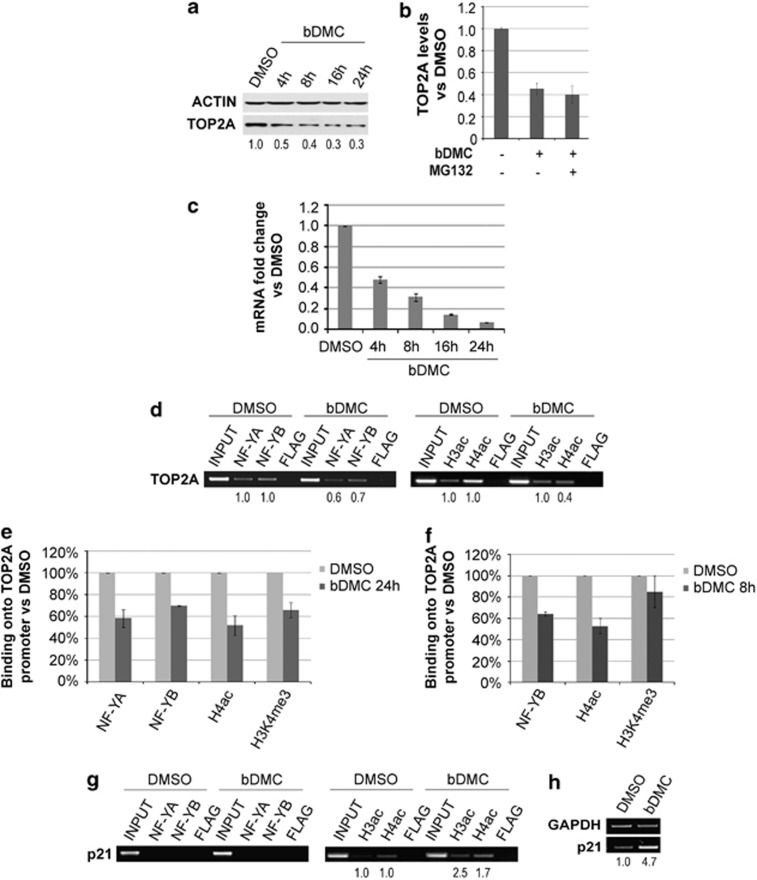
Inhibition of NF-Y-mediated TOP2A transcription by bDMC. (**a**) TOP2A protein analysis in MNase-treated extracts following time course incubation with bDMC (30 *μ*M). The indicated values represent the quantification of TOP2A immunoreactive bands of two independent experiments normalized to actin levels. (**b**) Quantification of TOP2A expression levels (normalized to actin) following bDMC and bDMC-MG132 administration for 24 h *versus* DMSO (arbitrarily set at 1). (**c**) Real-time analysis of TOP2A transcripts in cells treated with 30 *μ*M bDMC, represented as mRNA fold change *versus* DMSO (arbitrarily set at 1). GAPDH has been used as internal control. The indicated values are mean of four independent experiments ±S.D. (**d**) ChIP semi-quantitative PCR analysis of NF-YA, NF-YB, acetyl-H3 and acetyl-H4 binding to TOP2A promoter in cells treated with DMSO or bDMC (30 *μ*M) for 24 h. (**e** and **f**) ChIP real-time analysis of chromatin-associated NF-Y, acetyl-H4 and H3K4me3 to TOP2A promoter following 24-h (**e**) and 8-h (**f**) incubation with 30 *μ*M bDMC. (**g**) Semi-quantitative analysis of the recruitment of NF-Y and acetylated histones on p21 regulatory region cells treated for 24 h with DMSO and 30 *μ*M bDMC. (**h**) mRNA expression levels of p21 in 24 h bDMC-treated cells

Despite MNase digestion, a time-dependent decrease of TOP2A levels was observed following bDMC administration (Figure 5a). In addition, proteasome inhibition by MG132 did not raise TOP2A levels (Figure 5b), suggesting that neither the formation of TOP2A-DNA complexes or protein degradation were responsible for TOP2A halved levels. For this reason, we analyzed TOP2A mRNA levels after time course incubation with bDMC. Real-time RT-PCR analysis revealed that bDMC induced a clear time-dependent decrease of TOP2A gene transcription, with maximal inhibition after 24 h (Figure 5c). The release from bDMC for 16 and 24 h did not restore TOP2A mRNA and protein levels (Supplementary Figures S2a and b).

The transcription factor NF-Y is the key activator of TOP2A and specifically binds the CCAAT boxes of its regulatory regions. Chromatin immunoprecipitation (ChIP) experiments were performed to investigate NF-Y binding to TOP2A promoter after 24 h of bDMC administration. Compared with DMSO, the binding of NF-YA and NF-YB subunits was reduced by about 40 and 30%, respectively (Figure 5d). A strong correlation between NF-Y binding and epigenetic marks has been described, therefore we investigated by ChIP the levels of acetylated histones H3 and H4 (H3ac and H4ac) and H3K4me3, an epigenetic marker associated with active gene promoters, whose deposition is strongly dependent upon the binding of NF-Y.^47, 48^ Although H3ac was not modified by bDMC, the levels of H4ac were reduced by about 60% on the TOP2A promoter region (Figure 5d). Real-time PCRs highlighted that maximum NF-Y and H4ac reduction were induced after 8 h, while H3K4me3 showed a decrease at 24 h, hinting that NF-Y dissociation could lead to later effects on the chromatin structure of TOP2A promoter and therefore on gene transcription (Figures 5e and f). On the other hand, both H3 and H4 acetylation increased on p21 regulatory region, consistently to its transcriptional activation (Figures 5g and h). Taken together, these data indicate that bDMC induces the transcriptional repression of TOP2A by affecting NF-Y binding to its promoter region.

### bDMC affects NF-YA expression and sub-cellular localization of NF-YB and NF-YC subunits

**Figure 6:**
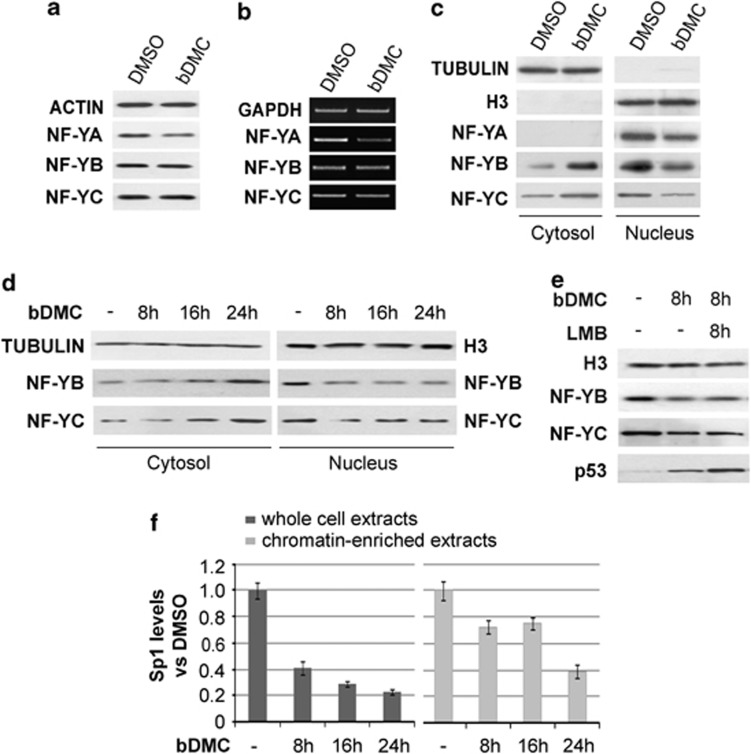
bDMC affects NF-Y subunits expression and sub-cellular localization. (**a**) Western blot analysis of total extracts of DMSO and bDMC (30 *μ*M)-treated cells (24 h) with antibodies against the three NF-Y subunits. (**b**) Semi-quantitative RT-PCRs of NF-YA, NF-YB and NF-YC subunits following 24 h treatment with DMSO and 30 *μ*M bDMC. (**c**) Expression levels of NF-YB and NF-YC in nuclear and cytosolic fractions of HCT116 cells treated with 30 *μ*M bDMC for 24 h. Tubulin and H3 were used as loading control for cytosolic and nuclear extracts, respectively. (**d**) Time course analysis of NF-Y subunits expression in nuclear and cytoplasmic cellular compartments. (**e**) Nuclear expression levels of NF-YB, NF-YC and p53 following co-incubation of 30 *μ*M bDMC with LMB for 8 h *versus* bDMC alone. Histone H3 was used as loading control. (**f**) Expression levels of SP1 in total and chromatin-enriched extracts following time course incubation with 30 *μ*M bDMC

We next wondered whether decreased NF-Y binding to TOP2A CCAAT boxes could be ascribed to reduced NF-Y cellular levels upon bDMC treatment. Total extracts were prepared from HCT116 cells treated with DMSO and bDMC for 24 h. Western blot analysis showed a decrease of the DNA binding subunit NF-YA, consistently with mRNA halved levels (Figures 6a and b); no changes were observed in NF-YB and NF-YC expression levels (Figures 6a and b). While NF-YA has been shown to be mainly localized in the nucleus, NF-YB and NF-YC shuttle from the cytoplasm to the nucleus.^49^ Therefore, we performed western blot analysis of NF-Y subunits on nuclear and cytosolic fractions of bDMC-treated cells. As shown in Figure 6c, NF-YB and NF-YC were accumulated in the cytoplasm. A time-dependent increase of cytosolic NF-YB and NF-YC was observed following bDMC administration, with a maximum peak after 24 h of treatment (Figure 6d), although nuclear NF-YB and NF-YC were reduced to minimum levels already after 8 h. To discriminate whether nuclear export rather than import was affected by bDMC, we co-treated cells with Leptomycin B (LMB), a known inhibitor of CRM1 exportin: no increase of nuclear NF-YB and NF-YC was observed in co-treated *versus* bDMC-treated cells (Figure 6e). The accumulation of nuclear p53 confirmed the efficacy of LMB treatment on HCT116 cells. These results support the hypothesis that bDMC induces Topo II*α* transcriptional repression by decreasing NF-YA levels and retaining NF-YB/NF-YC into the cytoplasm.

The transcriptional control of TOP2A gene has been shown to be played by NF-Y in cooperation with the transcription factor Sp1.^50^ For this reason, we decided to investigate whether also Sp1 could have a role in the time-dependent decrease of TOP2A mRNA levels. Chromatin-enriched and whole-cell extracts were prepared following 4, 8 16 and 24 h exposure of HCT116 cells to bDMC and western blot analysis performed with anti-Sp1 antibody. As shown in Figure 6f, while total Sp1 levels were already reduced at 8 h, bDMC mainly affected Sp1 chromatin recruitment following 24 h.

### Cell death induced by bDMC is mediated by TOP2*α* enzymatic and transcriptional targeting

**Figure 7:**
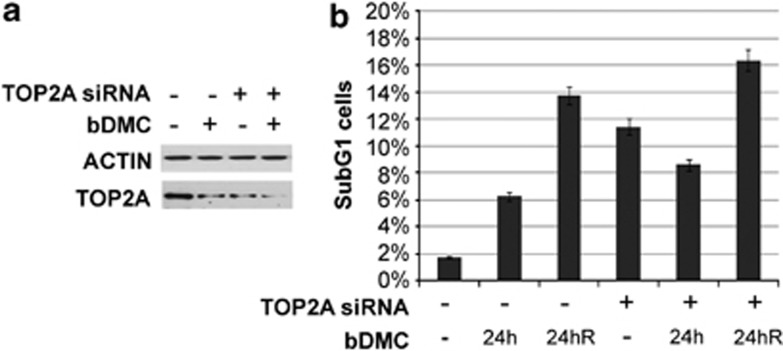
bDMC cytotoxic activity is mediated by Topoisomerases II*α* enzymatic and transcriptional targeting. (**a**) Western blot analysis of TOP2A following siRNA-mediated knockdown of TOP2A and bDMC (30 *μ*M) treatment for 24 h. (**b**) Quantification of SubG1 population upon 30 *μ*M bDMC treatment and release (R) in control and TOP2A-inactivated cells

To assess the importance of TOP2A in mediating bDMC apoptotic response, we knocked down TOP2A by transient RNAi. TOP2A protein levels were reduced by >70% (Figure 7a) and SubG1 events raised from 1.8 to 11.5% after small interfering RNA (siRNA) transfection for 48 h (Figure 7b), highlighting the fundamental role of TOP2A in HCT116 viability. Following bDMC administration, SubG1 events raised to 6.3% in control cells, while no increase was observed in TOP2A-deficient cells (from 11.5 to 8.7%), although TOP2A protein levels were further reduced (Figure 7a). On the other hand, the release from bDMC for additional 24 h resulted in twofold increase of apoptosis also in TOP2A knockdown cells (from 6.3 to 13.7% in control cells and from 8.6 to 16.4% in silenced cells).

These data suggest that both enzymatic and transcriptional TOP2A targeting contributes to bDMC cytotoxic activity. The lack of an additive apoptotic effect of bDMC administration on TOP2A-silenced cells, indicates that within the first 24 h an important role is played by the enzymatic inhibition of TOP2A. Reduced TOP2A levels avoid a further increase of apoptotic events. Differently, TOP2A-depleted cells are still committed to irreversible cell death when bDMC is removed, suggesting that long-term apoptotic effects can be ascribed to TOP2A transcriptional inhibition rather than its poisoning.

## Discussion

High TOP2A levels result in enhanced proliferation rates of many human malignancies and correlate with shortened patient survival.^51, 52, 53^ These types of tumors are most susceptible to TOP2-targeting chemotherapeutics. For these reasons, TOP2A expression is a double-edged sword: on one side, high TOP2A levels could indicate tumor aggressiveness and poor outcome, on the other side, this could determine a positive response to chemotherapeutic drugs targeting this enzyme.

Our results provide evidence that bDMC suppresses cell proliferation and induces apoptosis, at least in part, by targeting TOP2A, thus encompassing bDMC in the category of TOP2-based DNA-damaging agents. Indeed, bDMC reduces cell viability by inducing DNA breaks and a persistent and irreversible DNA damage response and inhibits TOP2A activity as highlighted by MNase band-depletion and cell-free TOP2A relaxation assays (Figure 4).

Interestingly, bDMC does not cause DNA damage-induced cell death in normal cells, which are less susceptible to bDMC, as highlighted by higher IC50 concentrations (Figure 2). Tumor selectivity of Curcumin and its derivatives has been already attributed to different mechanisms, such as lower drug uptake in healthy cells,^54^ rather than increased sensitivity of cancer cells due to lower glutathione levels or constitutive expression of NF-kB.^55^ Taking into account that tumor cells have normally higher expression of TOP2A compared with normal cells, our data suggest that one of the molecular basis for bDMC cancer cell selectivity and susceptibility could be dependent on the levels of TOP2A target protein.

TOP2A has a fundamental role in DNA replication, and it has been shown that DNA damage induced by TOP2A poisons is related to their interaction with DNA replication fork progression.^56^ Although bDMC delays the progression from early to late S phase,^36^ the majority of cells positive for *γ*H2AX following bDMC administration are accumulated in G2/M phase (Figure 3), and chromosome aberrations are observed in mitotic cells (Figure 3). DNA damage response and repair are activated in G2/M cells, as demonstrated by the clear reduction of the G2/M population following pre-treatment with the PI3K-kinases inhibitor WORT (Figure 3). Surprisingly, these cells escaping the G2/M DNA damage checkpoint are not driven to apoptosis but are accumulated in the next G0/G1 phase, suggesting that apoptosis is triggered by a persistent activation of the DNA damage response in G2/M cells.

Although 4- and 8-h treatment with bDMC induces a concurrent inhibition of transcription and activity of TOP2A, prolonged administration for 16 and 24 h results only in reduced gene transcription (Figures 4 and 5). The inability of long-term poisoning of TOP2A by bDMC could be due to the rapid metabolism of the molecule inside the cell. After 24 h, <0.5% of bDMC was detected inside the cells,^36^ and about 7.5% (±1.5) and 6.2% (±0.7) after 4 and 8 h, respectively (data not shown). These data strongly suggest that the main effects are induced by bDMC within 8 h, consistently with maximum TOP2A enzymatic inhibition and reduced NF-Y activity in 8 h, although the molecular mechanisms triggered by bDMC or its metabolites, leading to TOP2A transcriptional inhibition, could continue for longer time.

To shed light on the contribution of TOP2A poisoning rather than transcriptional inhibition on apoptosis activation, we silenced TOP2A through transient RNAi (Figure 7). TOP2A knockdown leads to about sixfold increase of SubG1 events, and bDMC treatment for 24 h is not able to further augment apoptosis, suggesting that direct TOP2A targeting contributes to bDMC pro-apoptotic activity. The inhibition of mRNA levels, and consequently of protein levels of TOP2A, has a major role in the irreversible activation of cell death, observed after drug removal at 24 h.

TOP2A transcriptional inhibition results from decreased recruitment of the transcription factor NF-Y to its regulatory regions (Figure 5), as a consequence of halved NF-YA nuclear levels and NF-YB/NF-YC cytoplasmic retention (Figure 6). Despite a reduction of only 40 and 30% in the binding of NF-YA and NF-YB, respectively, to TOP2A promoter, TOP2A gene transcription is strongly affected by bDMC. This result is consistent with our previous data observed in NF-YB-inactivated cells: although NF-YB knockdown induces a robust decrease of TOP2A levels, NF-YA and NF-YB binding were reduced by about 20% compared with control cells.^57^

Finally, we showed that bDMC induces decreased expression and chromatin recruitment of Sp1, important functional partner of NF-Y in the control of TOP2A gene transcription (Figure 6). These results are in agreement with (i) Sp1 downregulation observed upon NF-Y inactivation by gene expression profiling,^57^ and (ii) ChIP-seq analysis (ENCODE Data at UCSC Genome Browser) showing NF-Y binding to Sp1 regulatory regions. All together, these data suggest that NF-Y directly controls Sp1 transcription, and therefore, TOP2A transcriptional downregulation induced by bDMC can be ascribed to reduced NF-Y and, consequently, Sp1 levels.

In addition to the identification of TOP2A as one of the molecular targets of bDMC, our data highlight that NF-Y regulation can be an interesting approach in anti-cancer therapy, taking into consideration that (i) NF-Y is one of the transcription factors orchestrating oncogenic transcriptional changes,^58, 59^ and (ii) clinical studies correlated upregulated expression of NF-Y target genes to poor clinical prognosis in multiple types of cancer.^60^

## Materials and Methods

### Cell lines and treatments

Human colorectal carcinoma HCT116 cells were cultured in Iscove's Modified Dulbecco's Medium (IMDM) supplemented with 10% fetal calf serum (FCS). Human hepatic fetal epithelial WRL-68 cells and colon adenocarcinoma LOVO cells were maintained in Dulbecco's Modified Eagle Medium (DMEM) with 10% FCS. Primary human fibroblasts (HF) were grown in DMEM 10% FCS, with Glutamine (2 mM) and Gentamicin (55 mg/l). Doubling time has been estimated of about 16 h for HCT116, LOVO and WRL68 cells, and 24 h for HF cells.

bDMC (1,7-bis[4-hydroxyphenyl]hepta-1,6-diene-3,5-dione) has been synthesized as previously reported^36^ and added to warm medium at the indicated concentrations. DMSO (Sigma-Aldrich Srl, Milan, Italy) was used as control. For pharmacological inhibitions, cells were pre-treated for 1 h and then co-incubated with bDMC for the indicated times with 25 *μ*M Z-VAD-fmk (Enzo Life Sciences, Inc., Farmingdale, NY, USA), 10 *μ*M WORT (Enzo Life Sciences, Inc.), 1 *μ*M MG132 (Sigma-Aldrich Srl), 20 mM NAC (Sigma-Aldrich Srl) and 10 ng/ml LMB (Enzo Life Sciences, Inc.).

### Crystal Violet assay

The inhibition of proliferation was measured by Crystal Violet staining, and the concentration at which cellular growth is inhibited by 50% (IC50) was determined following 24 h treatment with bDMC, as previously reported.^54^ Briefly, the cell monolayer was fixed with methanol and stained with 0.05% Crystal Violet solution in 20% methanol for 1 h. After washes, cells were allowed to dry. The incorporated dye was solubilized in acidic isopropanol and determined spectrophotometrically at 540 nm wavelength. The extracted dye was proportional to cell number.

### Flow cytometric analysis

Cells were harvested after drug treatments at the indicated time points and DNA distribution analysis of propidium iodide (PI)-stained cells was performed by an Epics cytofluorimeter (Beckman Coulter Srl, Milan, Italy).^36^ Apoptotic cells were detected by FACS using Annexin V-PE conjugate (BD Biosciences, Becton Dickinson Italia, Milan, Italy) following the protocol of the manufacturer.

Indirect immunofluorescence staining was performed as previously described.^57^ Briefly, harvested cells were washed with PBS 1X, fixed in 1% formaldehyde for 10 min at 37 °C and post-fixed with 90% ethanol. After permeabilization with 0.25% Triton X-100 in PBS 1X for 5 min, cells were stained with anti-phospho-H2AX Ser139 (No. 05-636, Millipore Spa, Vimodrone, Italy; 1 : 25) o.n. at 4 °C and with rabbit anti-mouse FITC-conjugated secondary antibody (No. 0313, Dako Italia Spa, Milan, Italy; 1 : 50) for additional 2 h at 4 °C. Following RNAse A treatment for 40 min, cells were incubated with PI (30 mg/ml) for 30 min at 4 °C and analyzed by an Epics cytofluorimeter (Beckman Coulter Srl).

### Chromosome spreads

DMSO- and bDMC-treated cells were collected, swollen in hypotonic solution (75 mM KCl) and incubated for 10 min at room temperature. After two washes in Fix solution (3 : 1(vol/vol) methanol:acetic acid), the cellular suspension was dropped on a wet ice-cold slide and stained for 15 min in Giemsa stain (No. 32884, Sigma-Aldrich Srl). Slides were washed in distilled water, air-dried and mounted with DPX mountant (No. 44581, Sigma-Aldrich Srl). Chromosomes were analyzed with a Nikon Eclipse 90i microscope (Nikon Instruments Spa, Florence, Italy).

### Immunoblotting

Whole-cell protein extracts were prepared by resuspending cells into 1X SDS sample buffer (25 mM Tris–HCl pH 6.8, 1.5 mM EDTA, 20% glycerol, 2% SDS, 5% β-mercaptoethenol, 0.0025% Bromophenol blue). Nuclear and cytoplasmic extracts were obtained by resuspending cells in 200 *μ*l of Solution A (10 mM HEPES pH7.9, 10 mM KCl, 1.5 mM MgCl2, 0.34 M sucrose, 10% glycerol, protease and phosphatase inhibitors), adding 0.1% Triton X-100 and incubating cells for 10 min on ice. The supernatant containing cytoplasmic proteins was collected by centrifugation at 1300 × *g* for 5 min at 4 °C, and the remaining pellet (nuclei) was disrupted in 1X SDS sample buffer (as above). Chromatin-enriched extracts were prepared as previously described.^57^ For immunoblotting, equivalent amounts of cellular extracts were resolved by SDS-PAGE, electrotransferred to PVDF membrane (GE Healthcare Italia, Milan, Italy) and immunoblotted. The following primary antibodies were used: anti-NF-YB and anti-NF-YC purified rabbit polyclonal antibodies; anti-phospo-H2AX (sc-101696), anti-H3 (C16) (sc-8654), anti-p53 (DO1) (sc-126), anti-TOP2A (K19) (sc-5347), anti-PARP1 (F2) (sc-8007), anti-NF-YA (sc-10779), anti-Sp1 (sc-420 X) and anti-actin (I19) (sc-1616) from Santa Cruz Biotecnology, Inc. (Dallas, TX, USA); anti-phospho-ATM Ser1981 (No. 4526), anti-phospho-BRCA1 Ser1524 (No. 9009), anti-phospho-Chk1 Ser296 (No. 2349) from Cell Signaling Technology, Inc. (Danvers, MA, USA); and anti-*α*-tubulin (T6074) from Sigma-Aldrich Srl. Chemiluminescent detection reagent has been purchased from Millipore Spa (Luminata Classico and Forte Western HRP).

### Immuno-band depletion assay

Briefly, 6 × 10^5^ cells were lysed in 50 *μ*l of alkaline lysis solution (200 mMNaOH, 2 mM EDTA), and the lysate was neutralized by 8 *μ*l of neutralization buffer (1 M HCl, 600 mM Tris pH 8.0). The neutralized lysate was mixed with 6.6 *μ*l of 10 × MNase reaction buffer (50 mM MgCl_2_, 50 mM CaCl_2_, 5 mM DTT, 1 mM EDTA, 1 mM PMSF, protease inhibitors) and incubated or not with 60 units of MNase. After 20 min digestion, 60 *μ*l of 2 × SDS sample buffer were added to each sample, and the lysates were separated on 8% SDS-PAGE gels and immunoblotted.

### TOP2 decatenation assay

TOP2 activity was assayed *in vitro* through the Topoisomerse II assay kit (No. TG1001, TopoGEN Inc., Port Orange, FL, USA), following the instructions of the manufacturer. Nuclear extracts containing TOP2 activity were obtained from HCT116 cells following the suggestions of the manufacturer, and the ability to decatenate kDNA was analyzed in the presence of DMSO and bDMC. Briefly, decatenation assay was performed with 50 ng kDNA in a 10-*μ*l reaction mixture containing 50 mM Tris–HCl (pH 8.0), 150 mM NaCl, 1 0 mM MgCl2, 0.5 mM dithiothreitol, 2 mM ATP, 30 *μ*g/ml BSA with DMSO or 30, 60, 120 *μ*M of bDMC and 0.5 *μ*g of cell nuclear extract. Reactions were incubated at 37 °C for 30 min and stopped by adding 5 *μ*l of stop buffer (5% Sarkosyl, 0.125% bromphenol blue and 25% glycerol). Samples were loaded directly onto a 1% agarose gel containing ethidium bromide (0.5 *μ*g/ml). TOP2 activity was measured by the appearance of decatenated minicircular products and determined as the percentage of DMSO by ImageJ software (Image J, U.S. National Institutes of Health, Bethesda, MD, USA).

### RT-PCR

RNA was extracted from cells treated with DMSO and bDMC using the Purelink RNA mini kit (Invitrogen, Life Technologies Italia, Monza, Italy) according to the manufacturer's protocol, and 3 *μ*g of RNA were retro-transcribed with a Moloney murine leukemia virus reverse transcriptase (Promega Italia SrL, Milan, Italy). Semi-quantitative and quantitative real-time PCRs were performed with oligonucleotides designed to amplify the cDNA of: GAPDH (forward 5′-ACAGTCAGCCGCATCTTCTT-3′ reverse 5′-GCCCAATACGACCAAATCC-3′), TOP2A (forward 5′-TGGCAGAGGCAGAGAGAGTT-3′ reverse 5′-TCAAAAAGCACCATAGAGTTGC-3′) and p21 (forward: 5′-TGACCCTGAAGTGAGCACAG-3′ reverse: 5′-GGGAAAAGGCTCAACACTGA-3′). Semi-quantitative PCR results were analyzed by ImageJ software, whereas relative fold change enrichments of real-time PCR samples were calculated with the formula 2^−(ΔΔCt)^, where −(ΔΔCt)=−((Ct_target_−Ct_GAPDH_)_bDMC_−(Ct_target_−Ct_GAPDH_)_DMSO_).

### siRNA transfection

HCT116 cells were transfected (Metafectene SI^+^, Biontex Laboratories GmbH, Martinsried/Planegg, Germany) with 300 nM of paired TOP2A and non-targeting control siRNAs obtained from Sigma-Aldrich Srl. TOP2A sense strand sequence: 5′-AAGACTGTCTGTTGAAAGAA-3′. Cells were analyzed or treated with bDMC 36 h after transfection.

### ChIPs

ChIPs were performed as previously described.^54^ In all, 4 *μ*g of the following antibodies were added to each IP and incubated overnight at 4 °C on a rotating wheel: anti-H3Ac (No. 06-599, Millipore Spa), anti-H4Ac (No. 06-866, Millipore Spa), anti-H3K4me3 (No. ab8580, AbCam, Cambridge, UK), anti-NF-YA (No. sc-10779, Santa Cruz), anti-NF-YB purified polyclonal antibody and anti-FLAG (No. F7425, Sigma-Aldrich Srl), used as control for non-specific interactions. DNAs were resuspended in TE buffer, and PCR analyses were performed with the following primers: p21 (forward: 5′-GTAAATCCTTGCCTGCCAGAGTG-3′ reverse: 5′-GCTGCCCAGCGCCGAGCCAG-3′) and TOP2A (forward: 5′-CGTCAGAACAGAGGACAGTTTTT-3′ reverse 5′-TGGAAGAGATGGGCTTTGG-3′). Semi-quantitative PCR results were analyzed by ImageJ software. Relative fold change enrichments of real-time quantitative PCR samples were calculated with the formula 2^−(ΔΔCt)^, where −ΔΔCt=−((Ct_sample_−Ct_input_)_bDMC_−(Ct_sample_−Ct_input_)_DMSO_).

## Abbreviations

TOP2: Topoisomerase II
bDMC: bis-DemethoxyCurcumin
DSB: double-strand break
PI: propidium iodide
LMB: Leptomycin B
NAC: *N*-acetylcisteine
WORT: Wortmannin
