# Supplementary figures and images for: Concurrent inhibition of enzymatic activity and NF-Y-mediated transcription of Topoisomerase-IIα by bis-DemethoxyCurcumin in cancer cells
Source: Cell Death Dis. 2013 Aug 8;4(8):e756–. doi: 10.1038/cddis.2013.287 (PMC3763449; doi:10.1038/cddis.2013.287)

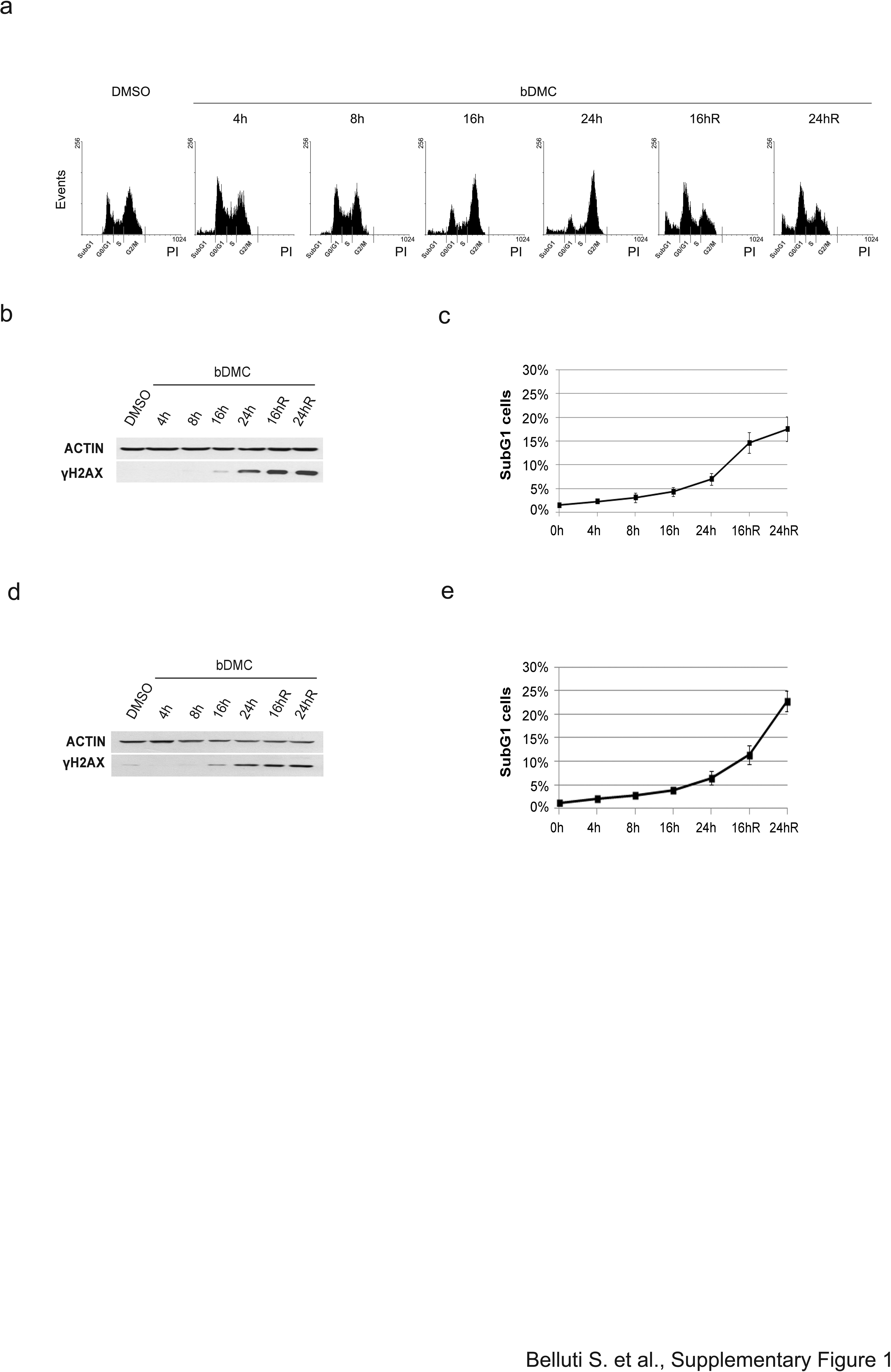

Supplement: Supplementary Figure 1 [file cddis2013287x1.tif]

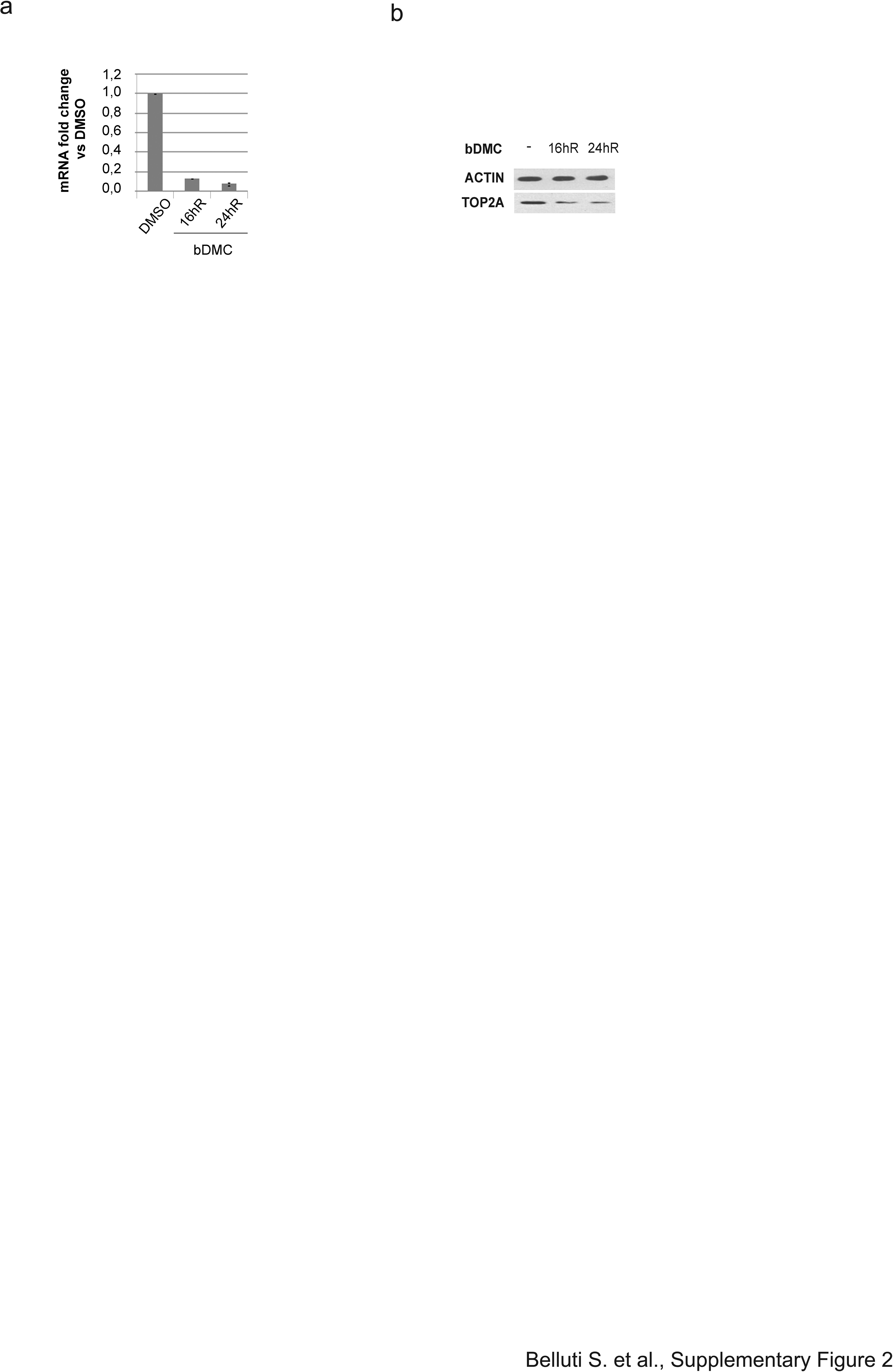

Supplement: Supplementary Figure 2 [file cddis2013287x2.tif]
