# Supplementary material for: Concurrent inhibition of enzymatic activity and NF-Y-mediated transcription of Topoisomerase-IIα by bis-DemethoxyCurcumin in cancer cells
Source: Cell Death Dis. 2013 Aug 8;4(8):e756–. doi: 10.1038/cddis.2013.287 (PMC3763449; doi:10.1038/cddis.2013.287)
Supplement: Supplementary Figures Legends [file cddis2013287x3.doc]

**Supplementary Figure 1.** bDMC apoptotic activity in LOVO and HCT116/E6 cells. (**a**) Cytofluorimetric cell cycle analysis of LOVO cells upon 30 μM bDMC treatment and release (R). (**b**) Expression levels of γH2AX in DMSO and 30 μM bDMC treated and released (R) LOVO cells. (**c**) Percentage of SubG1 events in LOVO cells following bDMC (30 μM) administration and release. (**d**) γH2AX Western blot analysis in HCT116/E6 cells treated with 30 μM bDMC and released for 16 or 24 hours (16hR and 24hR). (**d**) Changes in SubG1 population in HCT116/E6 cells following bDMC (30 μM) administration and release.

**Supplementary Figure 2.** Stable effect of bDMC on TOP2A expression. (**a**) *Real-Time* mRNA fold change of TOP2A expression *versus* DMSO (arbitrarily set at 100%) after 16 and 24 release (16hR and 24hR) from bDMC (30 μM) treatment for 24 hours. TOP2A levels have been normalized with GAPDH levels. (**b**) Western blot analysis of TOP2A protein expression after bDMC removal from culture medium for 16 and 24 hours (16hR and 24hR). Actin was used as loading control.
